# Impact of bowel preparation on elective colectomies for diverticulitis: analysis of the NSQIP database

**DOI:** 10.1186/s12876-022-02491-8

**Published:** 2022-09-12

**Authors:** Haoran Zhuo, Zheng Liu, Benjamin J. Resio, Jialiang Liu, Xishan Wang, Kevin Y. Pei, Yawei Zhang

**Affiliations:** 1 Department of Environmental Health Sciences, Yale School of Public Health, New Haven, CT 06511 USA; 2 Department of Colorectal Surgery, National Cancer Center/National Clinical Research Center for Cancer/Cancer Hospital, Chinese Academy of Medical Science and Peking Union Medical College, Beijing, 100021 China; 3 Department of Surgery, Yale School of Medicine, New Haven, CT 06520 USA; 4 Parkview Health Graduate Medical Education, Fort Wayne, IN 46805 USA; 5 Department of Cancer Prevention and Control, National Cancer Center/National Clinical Research Center for Cancer/Cancer Hospital, Chinese Academy of Medical Sciences and Peking Union Medical College, Beijing, 100021 China

**Keywords:** Bowel preparation; Diverticulitis; Colectomy; NSQIP

## Abstract

**Background:**

Recent data based on large databases show that bowel preparation (BP) is associated with improved outcomes in patients undergoing elective colorectal surgery. However, it remains unclear whether BP in elective colectomies would lead to similar results in patients with diverticulitis. The purpose of this study was to investigate whether bowel preparation affected the surgical site infections (SSI) and anastomotic leakage (AL) in patients with diverticulitis undergoing elective colectomies.

**Study design:**

We identified 16,380 diverticulitis patients who underwent elective colectomies from the American College of Surgeons National Surgical Quality Improvement Program (ACS-NSQIP) colectomy targeted database (2012–2017). Multivariate logistic regression models were employed to investigate the impact of different bowel preparation strategies on postoperative complications, including SSI and AL.

**Results:**

In the identified population, a total of 2524 patients (15.4%) received no preparation (NP), 4715 (28.8%) mechanical bowel preparation (MBP) alone, 739 (4.5%) antibiotic bowel preparation (ABP) alone, and 8402 (51.3%) MBP + ABP. Compared to NP, patients who received any type of bowel preparations showed a significantly decreased risk of SSI and AL after adjustment for potential confounders (SSI: *MBP* [OR = 0.82, 95%CI: 0.70–0.96], *ABP* [0.69, 95%CI: 0.52–0.92]; AL: *MBP* [OR = 0.66, 95%CI: 0.51–0.86], *ABP* [0.56, 95%CI: 0.34–0.93]), where the combination type of MBP + ABP had the strongest effect (SSI:OR = 0.58, 95%CI:0.50–0.67; AL:OR = 0.46, 95%CI:0.36–0.59). The significantly decreased risk of 30-day mortality was observed in the bowel preparation of MBP + ABP only (OR = 0.32, 95%CI: 0.13–0.79). After the further stratification by surgery procedures, patients who received MBP + ABP showed consistently lower risk for both SSI and AL when undergoing open and laparoscopic surgeries (Open: *SSI* [OR = 0.51, 95%CI: 0.37–0.69], *AL* [OR = 0.47, 95%CI: 0.25–0.91]; Laparoscopic: *SSI* [OR = 0.58, 95%CI: 0.47–0.72*, AL* [OR = 0.49, 95%CI: 0.35–0.68]).

**Conclusions:**

MBP + ABP for diverticulitis patients undergoing elective open or laparoscopic colectomies was associated with decreased risk of SSI, AL, and 30-day mortality. Benefits of MBP + ABP for diverticulitis patients underwent robotic surgeries warrant further investigation.

## Background

The role of bowel preparations prior to elective colectomies continues to be debated. Previous studies have suggested that oral antibiotic bowel preparation (ABP) and the combination of oral antibiotic and mechanical bowel preparation (ABP + MBP) before colectomy may decrease the risk of postoperative complications such as anastomotic leakage (AL), surgical site and deep space infections, and 30-day mortality [1–3]; while the effects of MBP alone remain controversial [4–9]. However, these previous studies were largely generated from colectomy patients for whom the indication for surgery was not distinguished, or from patients with colorectal cancer only. Information concerning the role of bowel preparation before elective colectomies for other bowel diseases, like diverticulitis, is lacking.

Diverticulitis is an increasingly common disease, accounting for nearly 300,000 hospital admissions and $1.8 billion of direct medical costs per year in the U.S. in the last decade [10]. Although nonsurgical treatment is largely considered for diverticulitis management, patients with certain diverticulitis conditions still require colectomy [10, 11]. Specifically, surgical treatment is considered among patients with recurrent chronic diverticulitis, and is effectively performed with low mortality rate for acute diverticulitis patients [10–13]. However, the postoperative morbidity and mortality rates of colectomy are higher among patients with diverticulitis compared to colorectal cancer [14, 15]. Bowel preparation is one measure to mitigate the postoperative complications of colectomy among colorectal cancer or all cases underwent surgery [16–18]. To date, there is very little published data investigating the role of BP prior to elective colectomy specifically among patients with diverticulitis. To sum up, it remains unclear whether BP with elective colectomies would have benefits among diverticulitis cases. The purpose of this study was to investigate the impact of bowel preparation on surgical site infection (SSI) and AL in patients with diverticulitis undergoing elective colectomy.

## Methods

### Study population

We conducted a retrospective cohort study using the general American College of Surgeons National Surgical Quality Improvement Program (ACS NSQIP) and colectomy targeted ACS NSQIP database from 2012 to 2017. The full details of data collection for NSQIP can be found elsewhere [19]. In brief, the NSQIP database is nationally validated, risk-adjusted, and outcomes-based programs to measure and improve the quality of surgical care, including prospective information on presurgical risk factors and 30-day postoperative mortality/morbidity outcomes from random sampling of multiple participating institutions. 16,723 individuals were eligible in our study with chronic diverticulitis, known types of bowel preparation and major outcomes (SSI, AL), and underwent elective colorectal resections. According to NSQIP, the use of MBP and ABP were separately recorded according to “yes,” “no,” or “unknown,” and MBP did not include enemas or suppositories and ABP did not include parenteral antibiotics [2]. Elective colorectal resections were identified by primary Current Procedural Terminology (CPT) codes (44140, 44145, 44160, 44204, 44205, and 44207). We excluded 343 Patients from analysis with urgent or emergency cases, preoperative ventilator dependent, renal failure, systematic sepsis, or classified by an American Society of Anesthesiologists (ASA) physical status of five (V), which was in order to remove potential non-elective surgeries.

### Covariates

Primary exposure was the type of bowel preparation that derived from the combination of MBP and ABP, with implementation of both types as ABP + MBP and individual use of ABP and MBP separately. Other covariates included preoperative characteristic of age, race/ethnicity, sex, body mass index (BMI), ASA classification, smoking status, non-independent functional status, diabetes, hypertension, history of congestive heart failure (CHF), chronic obstructive pulmonary disease (COPD), weight loss (> 10% loss of body weight in the last 6 months), bleeding disorder (including clotting deficiencies and long-term anticoagulation therapy that was not stopped before surgery) and steroid use, and intraoperative characteristics of resection type (left, right), wound classification (clean, clean contaminated, contaminated, dirty/infected) [20], approach type (open, laparoscopic, robotic), and operative time > 3 h.

### Outcome assessment

Primary outcomes were overall SSI rates (superficial, deep, and organ space), as defined by the U.S. CDC [21]; and AL was defined as any leak of endoluminal contents through an anastomosis. Secondary outcomes were 30-day mortality, wound dehiscence, infectious complications (wound infection, pneumonia, urinary tract infection, sepsis), pulmonary complications (pneumonia, pulmonary embolism, reintubation), cardiac complications (myocardial infarction and cardiac arrest), ileus, length of hospital stay (LOS) ≥ 4 days, and reoperations/readmissions.

### Statistical analysis

Included patients were divided into four groups based on the type of BP: no preparation (NP); ABP; MBP; MBP + ABP. We used Pearson’s Chi-squared test and ANOVA test to investigate patients’ demographics, preoperative comorbidities and intraoperative characteristics among the four groups of BP type. Multivariable logistic regression was used to examine the association between preoperative BP and both major and secondary outcomes in diverticulitis patients. The one-to-one greedy propensity score matching on the selected demographic characteristics (age, race/ethnicity) and preoperative risk factors (ASA classification, CHF, weight loss > 10%, preoperative steroid use) between each BP type (MBP alone, ABP alone, MBP + ABP) and NP was also used to examine the association between BP type and major outcomes. The role of BP was further investigated for major outcomes only (SSI, AL) among a stratified population that underwent open, laparoscopic, and robotic surgeries as well as patients underwent left- and right-sided colectomy. All significance tests were implemented in SAS 9.4 with a significance level of two-tailed *Type I error (α)* of 0.05.

## Results

Of the identified 16,380 diverticulitis patients who underwent elective colectomies, 2524 (15.4%) received NP, 4715 (28.8%) MBP alone, 739 (4.5%) ABP alone, and 8402 (51.3%) MBP + ABP. Patients who received NP were more likely to be non-whites (*P* < 0.0001), ASA III-IV (*P* = 0.011), to have experienced congestive heart failure (*P* = 0.046) and to have used preoperative steroids (*P* = 0.006) compared to those who received MBP alone, ABP alone, or MBP + ABP. Patients who received ABP alone were more likely to be younger than 55 years (*P* = 0.002) and to have lost weight  (*P* = 0.004) than others (Table 1).

**Table 1:** Demographic and preoperative characteristics of diverticulitis patients who underwent elective colectomy by type of bowel preparation

	Type of bowel preparation								
	NP		MBP alone		ABP alone		MBP+ABP		*P* value
	(N=2524)	(%)	(N=4715)	(%)	(N=739)	(%)	(N=8402)	(%)	
Age (years)									**0.022**
<55	981	38.87	1760	37.33	298	40.32	3176	37.80	
55–64	755	29.91	1412	29.95	214	28.96	2645	31.48	
65–74	546	21.63	1096	23.24	172	23.27	1907	22.70	
≥75	242	9.59	447	9.48	55	7.44	674	8.02	
Race/ethnicity									**<0.0001**
White	1909	75.63	3851	81.68	594	80.38	7021	83.56	
(row %)		14.27		28.79		4.44		52.49	
Black	181	7.17	266	5.64	42	5.68	472	5.62	
(row %)		18.83		27.68		4.37		49.12	
Hispanic	183	7.25	222	4.71	56	7.58	485	5.77	
(row %)		19.34		23.47		5.92		51.27	
Others/Unknown	251	9.94	376	7.97	47	6.36	424	5.05	
(row %)		22.86		34.24		4.28		38.62	
Sex									0.620
Male	1091	43.23	2063	43.75	322	43.57	3743	44.55	
Female	1433	56.77	2652	56.25	417	56.43	4659	55.45	
BMI (kg/m^2^)									0.399
<25	583	23.57	1036	22.29	160	21.83	1823	21.91	
25–29	862	34.84	1696	36.49	244	33.29	2958	35.55	
30–34	590	23.85	1111	23.90	188	25.65	2095	25.18	
≥35	439	17.74	805	17.32	141	19.24	1444	17.36	
ASA classification									**0.013**
1–2	1500	59.43	3000	63.63	465	62.92	5324	63.37	
3–4	1022	40.49	1709	36.25	273	36.94	3073	36.57	
Missing	2	0.08	6	0.13	1	0.14	5	0.06	
Current smoker	530	21.00	969	20.55	137	18.54	1602	19.07	0.057
Non-independent functional health status	28	1.11	76	1.61	8	1.08	125	1.49	0.295
Diabetes	254	10.06	457	9.69	77	10.42	830	9.88	0.914
Hypertension	1117	44.26	2102	44.58	326	44.11	3639	43.31	0.531
Congestive heart failure	12	0.48	18	0.38	1	0.14	16	0.19	**0.046**
COPD	93	3.68	169	3.58	21	2.84	274	3.26	0.512
Weight loss>10%	61	2.42	101	2.14	32	4.33	199	2.37	**0.004**
Bleeding disorder	47	1.86	80	1.70	11	1.49	140	1.67	0.884
Preoperative steroid	118	4.68	151	3.20	20	2.71	326	3.88	**0.006**

Bold values are significant results with two-side *P*-values <0.05

Among intraoperative characteristics, patients who received no bowel preparation before surgery were more likely to undergo open procedures and right resection, and least likely to have robotic approach. Patients with ABP alone were more likely to have undergone left resections, experienced wound contamination and had an operation time > 3 h (Table 2).

**Table 2:** Intraoperative characteristics of diverticulitis patients who underwent elective colectomy by type of bowel preparation

	Type of bowel preparation								
	NP		MBP alone		ABP alone		MBP + ABP		*P* value
	(N = 2524)	(%)	(N = 4715)	(%)	(N = 739)	(%)	(N = 8402)	(%)	
Type of resection									** < 0.0001**
Left	2437	96.55	4660	98.83	727	98.38	8277	98.51	
Right	87	3.45	55	1.17	12	1.62	125	1.49	
Wound classification									** < 0.0001**
Clean	19	0.75	36	0.76	9	1.22	42	0.50	
Clean/contaminated	1734	68.70	3488	73.98	479	64.82	5860	69.75	
Contaminated	446	17.67	701	14.87	148	20.03	1546	18.40	
Dirty/infected	325	12.88	490	10.39	103	13.94	954	11.35	
Approach									** < 0.0001**
Open	439	25.27	515	14.92	86	15.66	1043	16.74	
Laparoscopic	1524	70.04	3006	75.78	455	71.65	5149	70.79	
Robotic	213	9.79	446	11.24	94	14.80	1082	14.87	
Operative time > 3 h	1256	49.76	2328	49.37	407	55.07	4285	51.00	**0.019**

Bold values are significant results with two-side *P*-values <0.05

The overall incidence rates of SSI and AL were 8.55% and 2.74%, respectively. Patients who received MBP + ABP had significantly lower rates of SSI and AL than the other BP types (Table 3). Similarly, the incidence rates for secondary outcomes including 30-day mortality, wound dehiscence, infectious complications, and reoperation were lower among patients received MBP + ABP compared to other bowel preparation types.

**Table 3:** Crude complication rates of primary and secondary outcomes by type of bowel preparation

	Type of bowel preparation										
	Overall		NP		MBP alone		ABP alone		MBP + ABP		*P* value
	N = 16,380	%	(N = 2524)	(%)	(N = 4715)	(%)	(N = 739)	(%)	(N = 8402)	(%)	
SSI	1400	8.55	298	11.81	458	9.71	62	8.39	582	6.93	** < 0.0001**
Anastomotic leak*	441	2.74	111	4.55	139	2.98	19	2.61	172	2.08	** < 0.0001**
30-day mortality	34	0.21	10	0.40	12	0.25	2	0.27	10	0.12	**0.030**
Wound dehiscence	101	0.62	25	0.99	38	0.81	4	0.54	34	0.40	**0.002**
Infectious complication	316	1.93	60	2.38	87	1.85	25	3.38	144	1.71	**0.004**
*Pulmonary complication*	131	0.80	30	1.19	40	0.85	4	0.54	57	0.68	0.068
*Cardiac complication*	56	0.34	12	0.48	14	0.30	1	0.14	29	0.35	0.470
Ileus	1200	7.33	231	9.15	378	8.02	45	6.09	546	6.50	** < 0.0001**
LOS ≥ 4 days	9794	59.79	1599	63.35	3067	65.05	421	56.97	4707	56.02	** < 0.0001**
Reoperation	590	3.60	119	4.71	200	4.24	25	3.38	246	2.93	** < 0.0001**
Readmission	1216	7.42	227	8.99	376	7.97	37	5.01	576	6.86	** < 0.0001**

Bold values are significant results with two-side *P*-values <0.05

Complications were defined as follows: pulmonary complications included pneumonia, pulmonary embolism, and reintubation; infectious complications included wound infection, pneumonia, urinary tract infection, and sepsis; cardiac complications included myocardial infarction and cardiac arrest, SSI was defined as superficial incisional, deep incisional, or organ space infections

*Include patients underwent anastomosis only

### Primary outcomes

After adjustment for baseline demographics and pre- and intra-operative characteristics, patients who received any type of BP had significantly lower risk of SSI and AL compared to those without BP (SSI: *MBP* [OR = 0.82, 95%CI: 0.70–0.96], *ABP* OR = 0.69, 95%CI: 0.52–0.92]; AL: *MBP* [OR = 0.66, 95%CI: 0.51–0.86], *ABP* [OR = 0.56, 95%CI: 0.34–0.93]), where the combination type of MBP + ABP had the strongest effect (SSI: OR = 0.58, 95%CI: 0.50–0.67; AL:OR = 0.46, 95%CI: 0.36–0.59) (Table 4). In a smaller sample of comparable populations that were matched on selected demographic characteristics and preoperative risk factors using propensity score, we observed similar associations between bowel preparations and better outcomes and SSI and AL except that the association between ABP and SSI was not significant.

**Table 4:** Multivariable logistic regression and propensity score (PS) matching^b^ to investigate the effect of different bowel preparation types on primary and secondary outcomes

	Overall	NP (REF)	MBP				ABP				MBP + ABP			
	N	N	N	OR*	95% CI*	*P*-value*	N	OR*	95% CI*	*P*-value*	N	OR*	95% CI*	*P*-value*
SSI	1400	298	458	**0.82**	**(0.70, 0.96)**	**0.015**	62	**0.69**	**(0.52, 0.92)**	**0.012**	582	**0.58**	**(0.50, 0.67)**	** < 0.0001**
(PS matching model ^b^)	Matched sample		**298/248**	**0.81**	**(0.68, 0.97)**	**0.024**	65/62	0.95	(0.66, 1.37)	0.781	**298/177**	**0.56**	**(0.46, 0.49)**	**<0.0001**
Anastomotic leak^a^	441	111	139	**0.66**	**(0.51, 0.86)**	**0.002**	19	**0.56**	**(0.34, 0.93)**	**0.024**	172	**0.46**	**(0.36, 0.59)**	** < 0.0001**
(PS matching model ^b^)	Matched sample		**111/78**	**0.66**	**(0.49, 0.89)**	**0.006**	**33/19 **	**0.57 **	**(0.32, 1.00)**	**0.050**	**111/62**	**0.52 **	** (0.38, 0.72) **	**<0.0001**
30-day mortality	34	10	12	0.61	(0.26, 1.44)	0.260	2	0.77	(0.17, 3.56)	0.737	10	**0.32**	**(0.13, 0.79)**	**0.013**
Wound dehiscence	101	25	38	0.80	(0.48, 1.33)	0.391	4	0.54	(0.19, 1.56)	0.253	34	**0.42**	**(0.25, 0.70)**	**0.001**
*Infectious complication*	316	60	87	0.79	(0.57, 1.11)	0.179	25	1.50	(0.93, 2.42)	0.101	144	0.77	(0.57, 1.05)	0.140
Pulmonary complication	131	30	40	0.70	(0.43, 1.13)	0.145	4	0.47	(0.16, 1.34)	0.158	57	**0.59**	**(0.38, 0.93)**	**0.022**
*Cardiac complication*	56	12	14	0.60	(0.27, 1.30)	0.194	1	0.29	(0.04, 2.23)	0.233	29	0.75	(0.38, 1.49)	0.417
Ileus	1200	231	378	0.89	(0.75, 1.06)	0.194	45	**0.67**	**(0.48, 0.94)**	**0.019**	546	**0.73**	**(0.62, 0.85)**	** < 0.001**
LOS ≥ 4 days	9794	1599	3067	**1.13**	**(1.02, 1.25)**	**0.021**	421	**0.80**	**(0.67, 0.95)**	**0.010**	4707	**0.78**	**(0.71, 0.85)**	** < 0.0001**
Reoperation	590	119	200	0.91	(0.72, 1.15)	0.419	25	0.72	(0.46, 1.12)	0.146	246	**0.62**	**(0.50, 0.78)**	** < 0.0001**
Readmission	1216	227	376	0.90	(0.76, 1.08)	0.255	37	**0.55**	**(0.38, 0.78)**	**0.001**	576	**0.77**	**(0.66, 0.91)**	**0.002**

Bold values are significant results with two-side *P*-values<0.05

^a^Include patients underwent anastomosis

^b^One-to-one greedy propensity score matching on age, race/ethnicity, ASA classification, history of congestive heart failure (CHF), weight loss (> 10% loss of body weight in the last 6 months), preoperative steroid use (Note: the sample size might be smaller due to the inability to find comparable observations on al matched variables)

*Adjusted for demographic characteristics (age, race/ethnicity, sex, body mass index (BMI), smoking status), preoperative comorbidities (ASA classification, non-independent functional status, diabetes, hypertension, history of congestive heart failure (CHF), chronic obstructive pulmonary disease (COPD), weight loss (> 10% loss of body weight in the last 6 months), bleeding disorder, steroid use), and intraoperative conditions (resection type, wound classification, approach type, operative time > 3 h)

LOS: length of total hospital stays (day)

After the further stratification of the general population by surgery procedure, patients who received MBP + ABP showed consistently lower risk for both SSI and AL among open and laparoscopic surgeries (SSI: *open* [OR = 0.51, 95%CI:0.37–0.69], *laparoscopic* [OR = 0.58, 95%CI:0.47–0.72]; AL: *open* [OR = 0.47, 95%CI:0.25–0.91], *laparoscopic* [OR = 0.49, 95%CI:0.35–0.68]). The significantly decreased rate of SSI was also observed among patients with ABP alone who underwent open procedures (OR = 0.49, 95%CI: 0.23–0.98). MBP alone was associated with a decreased rate of AL among laparoscopic procedures (OR = 0.69, 95%CI: 0.49–0.97). Little evidence of decreased risk of SSI and AK was observed among patients received bowel preparations before robotic colectomy (Table 5).

**Table 5:** Stratified multivariable logistic regression by surgical procedure and anatomic resection to investigate the effect of different bowel preparation types on primary outcomes

	NP	MBP			ABP			MBP + ABP		
	(ref)	OR*	95% CI*	*P*-value*	OR*	95% CI*	*P*-value*	OR*	95% CI*	*P*-value*
*Surgical procedure*										
Open procedure	*N* = *840*		*N* = *781*			*N* = *138*			*N* = *1468*	
SSI	ref	0.77	(0.55, 1.08)	0.131	**0.49**	**(0.23, 0.98)**	**0.044**	**0.51**	**(0.37, 0.69)**	** < 0.0001**
Anastomotic leak^a^	ref	0.71	(0.36, 1.40)	0.316	0.52	(0.12, 2.33)	0.394	**0.47**	**(0.25, 0.91)**	**0.024**
Laparoscopic procedure			*N* = *3942*			*N* = *626*			*N* = *6451*	
SSI	ref	0.81	(0.64, 1.02)	0.067	0.70	(0.46, 1.05)	0.084	**0.58**	**(0.47, 0.72)**	** < 0.0001**
Anastomotic leak^a^	ref	**0.69**	**(0.49, 0.97)**	**0.035**	0.60	(0.31, 1.17)	0.133	**0.49**	**(0.35, 0.68)**	** < 0.0001**
Robotic procedure			*N* = *559*			*N* = *115*			*N* = *1288*	
SSI	ref	1.40	(0.66, 2.96)	0.385	0.94	(0.28, 3.15)	0.926	1.02	(0.51, 2.06)	0.955
Anastomotic leak^a^	ref	0.70	(0.24, 2.08)	0.518	0.31	(0.04, 2.71)	0.291	0.58	(0.22, 1.53)	0.274
*Resection location*										
Left-sided resection	*N* = *2437*		*N* = *4660*			*N* = *727*			*N* = *8277*	
SSI	ref	0.87	(0.74, 1.02)	0.088	**0.68**	**(0.51, 0.92)**	**0.012**	**0.60**	**(0.51, 0.70)**	** < 0.0001**
Anastomotic leak^a^	ref	**0.68**	**(0.52, 0.87)**	**0.003**	**0.58**	**(0.35, 0.95)**	**0.029**	**0.47**	**(0.37, 0.60)**	** < 0.0001**
Right-sided resection	*N* = *87*		*N* = *55*			*N* = *12*			*N* = *125*	
SSI	ref	0.97	(0.32, 2.89)	0.949	1.25	(0.23, 6.86)	0.801	0.46	(0.18, 1.17)	0.103
Anastomotic leak ^b^	ref	–	–	–	–	–	–	–	–	–

Bold values are significant results with two-side *P*-values<0.05

^a^Patients underwent anastomosis

^b^No patients who underwent right-sided resection had anastomotic leak

*Adjusted for demographic characteristics (age, race/ethnicity, sex, body mass index (BMI), smoking status), preoperative comorbidities (ASA classification, non-independent functional status, diabetes, hypertension, history of congestive heart failure (CHF), chronic obstructive pulmonary disease (COPD), weight loss (> 10% loss of body weight in the last 6 months), bleeding disorder, steroid use), and intraoperative conditions (resection type (not included in the stratified analyses by resection location), wound classification, approach type (not included in the stratified analyses by surgical procedure), operative time > 3 h)

For different resection locations, the strongest effect of reduced risk of SSI and AL was observed using the combination type of MBP + ABP among patient with left resections (SSI: OR = 0.60, 95%CI: 0.51–0.70, AL: OR = 0.47, 95%CI: 0.37–0.60). The significantly decreased risk of SSI and AL was also observed among patients with ABP alone underwent left resections (SSI: OR = 0.68, 95%CI: 0.51–0.92; AL: OR = 0.58, 95%CI:0.35–0.95). MBP alone remained significantly associated with a decreased rate of AL among patients with left resections (OR = 0.68, 95%CI: 0.52–0.87). Limited evidence was found among right resection due to small sample size (Table 5).

### Secondary outcomes

Patients who received MBP + ABP had significantly decreased risk of secondary outcomes, including 30-day mortality (OR = 0.32, 95%CI: 0.13–0.79), wound dehiscence (OR = 0.42, 95%CI: 0.25–0.70), pulmonary complications (OR = 0.59, 95%CI: 0.38–0.93), ileus (OR = 0.73, 95%CI: 0.62–0.85), LOS ≥ 4 days (OR = 0.78, 95%CI: 0.71–0.85), reoperation (OR = 0.62, 95%CI: 0.50–0.78), and readmission (OR = 0.77, 95%CI: 0.66–0.91) compared to those without BP. Similarly, patients with ABP alone also had decreased risk of ileus (OR = 0.67, 95%CI: 0.48–0.94), LOS ≥ 4 days (OR = 0.80, 95%CI: 0.67–0.95), and readmission (OR = 0.55, 95%CI:0.38–0.78) compared to NP. On the contrary, preoperative MBP was associated with increased risk of LOS ≥ 4 days (OR = 1.13, 95%CI:1.02–1.25) compared to NP.

## Discussion

In this study, we found that patients who received any type of BP (MBP, ABP, MBP + ABP) had a significantly reduced risk of SSI and AL compared to NP, with the strongest association with the utility of combined mechanical and antibiotic preparation. This benefit of combination type of MBP + ABP was similar among open and laparoscopic surgeries, as well as left-side resections. In addition, combination type of MBP + ABP was significantly associated with decreased risk of secondary outcomes (30-day mortality, wound dehiscence, pulmonary complications, ileus, bleeding requiring transfusion, LOS ≥ 4 days, reoperation, and readmission) compared to NP. Evidence strongly supported that the combination type of MBP + ABP before colectomy is associated with reduced postoperative complications among diverticulitis patients.

The impact of bowel preparation regimens in patients underwent elective colectomy for diverticulitis is unknown. Although a Dutch study indicated that MBP alone prior to elective colorectal surgery for diverticulitis failed to decrease the AL rate and other septic complications compared to no bowel preparation [22], our study suggested that MBP alone was associated with decreased rate of SSI and AL in the general population. However, neither ABP alone or MBP + ABP were mentioned in the Dutch study and the results were limited by a relatively small sample size and the consideration of open procedures and left-sided colorectal surgery only. In the present study, we included a larger sample size which allowed for stratified analysis based on surgical approach (open, laparoscopic, robotic) and extent of anatomic resection (left, right). In our stratified analyses, we confirmed that MBP alone failed to decrease the risk of SSI and AL in open procedures but observed a significantly decreased risk of AL in laparoscopic procedures and left-sided resections for diverticulitis. In theory, MBP is believed to significantly reduce the bacterial burden, thereby protecting patients from postoperative anastomotic and infectious complications [23]. Laparoscopic procedures were also recommended as more reliable and efficacious forms of modality than open procedures due to shorter operative time and reduced blood loss for chronic diverticular disease [24]. Although the beneficial effects of MBP alone among colorectal cancer cases is controversial [4–9], this study demonstrated that MBP alone was associated with the decreased complication of AL when compared to no preparation.

The benefits of combination type MBP + ABP are superior to any preparation strategy that has been widely reported [1, 2, 25, 26]. Such combination preparation is physiologically sound: ABP reduces the bacterial concentration of the colonic mucosa while MBP improves antibiotic efficacy by reducing fecal bulk [27]. Our study also suggested that MBP + ABP was consistently associated with decreased rates of against postoperative complications and mortality among diverticulitis cases. The effect size of the association from MBP + ABP was strongest among all bowel preparations for the general population and a stratified population that underwent open and laparoscopic procedures. Little evidence in our study showed that bowel preparations before robotic colectomy was associated with decreased rates of any complications or mortality. It is possible that the small sample size of robotic procedures prevented us from finding significant results. Another possibility for the nonsignificant results might be the counteraction between the advantages of robotic colectomy on short-term outcomes and the negative impact of no bowel preparation [28]. Additional studies regarding the effects of any type of bowel preparation on robotic colectomies are needed.

Diverticulitis in Western countries is commonly left-sided while the right-sided is rare. The similar skewed distribution with dominant left-sided resections (left: 98.1%, right: 1.9%) was also observed in our study population. The benefits of bowel preparation, especially MBP + ABP, prior to elective colectomy among diverticulitis was observed in left-sided resections, while the limited sample size of right-sided resections precluded us from observing any significant outcomes among patient with right colectomy.

Protective effects of ABP alone on colorectal cancer have been identified for postoperative infectious complications, length of hospital stay, and readmission [29, 30]. For diverticulitis, our study found that ABP alone provided similar protective effects on SSI, AL, LOS ≥ 4 days, and readmission. In stratified analyses, ABP alone was not found to be associated with a reduced risk of infectious complications in laparoscopic procedures, but ABP alone was observed to significantly associated with decreased complication of SSI in open colectomies. However, our study was limited by a small number of ABP alone patients and more studies with larger sample sizes are needed to further confirm our findings.

There were several limitations to our study. First, the ACS-NSQIP database does not include historical information such as other previous treatment for diverticulitis, the number of episodes, as well as the prior administration of systemic antibiotics or parenteral antibiotics for each patient, which may be related to the outcomes of surgery. Nowadays, parenteral antibiotics is serving as routinely received preparation before colectomy [31]. As no evidence in NSQIP suggested a systematic difference of parenteral antibiotics receiving among patients received MBP, ABP or MBP + ABP, disregarding the potential implementation of parenteral antibiotics would induce relatively conservative effect size of BP in our study as the risk of AK and SSI would otherwise be higher if no parenteral antibiotics were received. Second, patients who underwent bowel preparation, particularly mechanical, were healthier suggesting that patient who received no bowel prep were potentially selected due to inability to tolerate the regimen. However, preoperative patient characteristics were adjusted in the multivariable regression analysis to minimize the potentiality of selection bias. Third, different practice patterns of surgeons might be another confounder, which is possible that the same surgeons who did not proceed bowel preparation for elective cases were essentially those with worse outcomes. However, the findings that bowel preparation had decreased risk of complication rates and mortality were preserved across different surgical procedures. It is likely that our results in the US cohort might not be generalizable to other populations or countries. Studies from distinct regions have observed different results of bowel preparations in the clinical practice and currently there are no “gold standard” practical guidelines with respect to it [32–34]. Further studies using different study population are therefore warranted to verify our results. Finally, we cannot exclude the possibility of residual confounding effect from other variables that were untracked by the database having influenced our results.

Despite these limitations, we have found that bowel preparation for chronic diverticulitis patients undergoing elective colectomies was associated with the decreased risk of postoperative complications and mortality. Our results were based on an observational cohort and the observed associations might not be causal effect. The observed associations should therefore be interpreted with caution and verified by further studies before considered into the clinical practice.

## Conclusion

Implementation of bowel preparation prior to an elective colectomy was successful in reducing SSI and AL among diverticulitis patients. The combination of ABP and MBP had the strongest effect reducing the risk of SSI, AL, and 30-day mortality in patients underwent open and laparoscopic procedures, suggesting that MBP + ABP might be a preferred bowel preparation type in cases of elective colorectal surgery for diverticulitis. Future randomized prospective trials are warranted to investigate the impact of bowel preparation, especially among robotic colectomies, and to provide more definitive answers.

## Data Availability

The data used is from the ACS-NSQIP and clinical records created by care providers is freely available to all institutional members who comply with the ACS-NSQIP data use agreement. The list and definitions of variables collected in the database can be found at the ACS-NSQIP website (https://www.facs.org/quality-programs/acs-nsqip).

## References

[1] Toh JWT, Phan K, Hitos K (2018). Association of mechanical bowel preparation and oral antibiotics before elective colorectal surgery with surgical site infection: a network meta-analysis. JAMA Netw Open.

[2] Kiran RP, Murray AC, Chiuzan C, Estrada D, Forde K (2015). Combined preoperative mechanical bowel preparation with oral antibiotics significantly reduces surgical site infection, anastomotic leak, and ileus after colorectal surgery. Ann Surg.

[3] Morris MS, Graham LA, Chu DI, Cannon JA, Hawn MT (2015). Oral antibiotic bowel preparation significantly reduces surgical site infection rates and readmission rates in elective colorectal surgery. Ann Surg.

[4] Bretagnol F, Panis Y, Rullier E (2010). Rectal cancer surgery with or without bowel preparation: the French GRECCAR III multicenter single-blinded randomized trial. Ann Surg.

[5] Bucher P, Gervaz P, Soravia C, Mermillod B, Erne M, Morel P (2005). Randomized clinical trial of mechanical bowel preparation versus no preparation before elective left-sided colorectal surgery. Br J Surg.

[6] Burke P, Mealy K, Gillen P, Joyce W, Traynor O, Hyland J (1994). Requirement for bowel preparation in colorectal surgery. Br J Surg.

[7] Saha AK, Chowdhury F, Jha AK, Chatterjee S, Das A, Banu P (2014). Mechanical bowel preparation versus no preparation before colorectal surgery: a randomized prospective trial in a tertiary care institute. J Nat Sci Biol Med.

[8] Zmora O, Mahajna A, Bar-Zakai B (2006). Is mechanical bowel preparation mandatory for left-sided colonic anastomosis? Results of a prospective randomized trial. Tech Coloproctol.

[9] Guenaga KF, Matos D, Wille-Jorgensen P (2011). Mechanical bowel preparation for elective colorectal surgery. Cochrane Database Syst Rev.

[10] Regenbogen SE, Hardiman KM, Hendren S, Morris AM (2014). Surgery for diverticulitis in the 21st century: a systematic review. JAMA Surg.

[11] Jurowich CF, Germer CT (2015). Elective surgery for sigmoid diverticulitis—indications, techniques, and results. Viszeralmedizin.

[12] Peppas G, Bliziotis IA, Oikonomaki D, Falagas ME (2007). Outcomes after medical and surgical treatment of diverticulitis: a systematic review of the available evidence. J Gastroenterol Hepatol.

[13] Rafferty J, Shellito P, Hyman NH, Buie WD (2006). Practice parameters for sigmoid diverticulitis. Dis Colon Rectum.

[14] Bokey EL, Chapuis PH, Pheils MT (1981). Elective resection for diverticular disease and carcinoma. Comparison of postoperative morbidity and mortality. Dis Colon Rectum.

[15] Oomen JLT, Cuesta MA, Engel AF (2007). Comparison of outcome of POSSUM, p-POSSUM, and cr-POSSUM scoring after elective resection of the sigmoid colon for carcinoma or complicated diverticular disease. Scand J Gastroenterol.

[16] Frazee RC, Roberts J, Symmonds R, Snyder S, Hendricks J, Smith R (1992). Prospective, randomized trial of inpatient vs. outpatient bowel preparation for elective colorectal surgery. Dis Colon Rectum.

[17] Solla JA, Rothenberger DA (1990). Preoperative bowel preparation—A survey of colon and rectal surgeons. Dis Colon Rectum.

[18] Jagelman DG, Fazio VW, Lavery IC, Weakley FL (1985). A prospective, randomized, double-blind study of 10% mannitol mechanical bowel preparation combined with oral neomycin and short-term, perioperative, intravenous Flagyl as prophylaxis in elective colorectal resections. Surgery.

[19] Khuri SF, Daley J, Henderson W (1998). The department of Veterans Affairs' NSQIP: the first national, validated, outcome-based, risk-adjusted, and peer-controlled program for the measurement and enhancement of the quality of surgical care. National VA surgical quality improvement program. Ann Surg.

[20] Centers for Disease Control and Prevention (CDC). Surgical site infection event (SSI). Accessed July, 2022. https://www.cdc.gov/nhsn/pdfs/pscmanual/9pscssicurrent.pdf.

[21] Horan TC, Gaynes RP, Martone WJ, Jarvis WR, Emori TG (1992). CDC definitions of nosocomial surgical site infections, 1992: a modification of CDC definitions of surgical wound infections. Infect Control Hosp Epidemiol.

[22] Van't Sant HP, Slieker JC, Hop WCJ (2012). The influence of mechanical bowel preparation in elective colorectal surgery for diverticulitis. Tech Coloproctol.

[23] Fa-Si-Oen PR, Verwaest C, Buitenweg J (2005). Effect of mechanical bowel preparation with polyethyleneglycol on bacterial contamination and wound infection in patients undergoing elective open colon surgery. Clin Microbiol Infect.

[24] Blake MF, Dwivedi A, Tootla A, Tootla F, Silva YJ (2005). Laparoscopic sigmoid colectomy for chronic diverticular disease. JSLS: J Soc Laparoendosc Surg.

[25] Klinger AL, Green H, Monlezun DJ (2019). The role of bowel preparation in colorectal surgery: results of the 2012–2015 ACS-NSQIP data. Ann Surg.

[26] Scarborough JE, Mantyh CR, Sun Z, Migaly J (2015). Combined mechanical and oral antibiotic bowel preparation reduces incisional surgical site infection and anastomotic leak rates after elective colorectal resection: an analysis of colectomy-targeted ACS NSQIP. Ann Surg.

[27] Ohman KA, Wan L, Guthrie T (2017). Combination of oral antibiotics and mechanical bowel preparation reduces surgical site infection in colorectal surgery. J Am Coll Surg.

[28] Benlice C, Aytac E, Costedio M (2017). Robotic, laparoscopic, and open colectomy: a case-matched comparison from the ACS-NSQIP. Int J Med Robotics Comput Assist Surg: MRCAS.

[29] Toneva GD, Deierhoi RJ, Morris M (2013). Oral antibiotic bowel preparation reduces length of stay and readmissions after colorectal surgery. J Am Coll Surg.

[30] Atkinson SJ, Swenson BR, Hanseman DJ (2015). In the absence of a mechanical bowel prep, does the addition of pre-operative oral antibiotics to parental antibiotics decrease the incidence of surgical site infection after elective segmental colectomy?. Surg Infect.

[31] Englesbe MJ, Brooks L, Kubus J (2010). A statewide assessment of surgical site infection following colectomy: the role of oral antibiotics. Ann Surg.

[32] Zmora O, Wexner SD, Hajjar L (2003). Trends in preparation for colorectal surgery: survey of the members of the American society of colon and rectal surgeons. Am Surg.

[33] Liu Z, Yang M, Zhao Z-X (2018). Current practice patterns of preoperative bowel preparation in colorectal surgery: a nation-wide survey by the Chinese society of colorectal cancer. World J Surg Oncol.

[34] Devane LA, Proud D, O'Connell PR, Panis Y (2017). A European survey of bowel preparation in colorectal surgery. Colorectal Dis.

